# Increasing Rubisco as a simple means to enhance photosynthesis and productivity now without lowering nitrogen use efficiency

**DOI:** 10.1111/nph.20298

**Published:** 2024-12-17

**Authors:** Coralie E. Salesse‐Smith, Yu Wang, Stephen P. Long

**Affiliations:** ^1^ Carl R. Woese Institute for Genomic Biology University of Illinois at Urbana‐Champaign Urbana IL 61801 USA; ^2^ DOE Center for Advanced Bioenergy and Bioproducts Innovation Urbana IL 61801 USA; ^3^ School of Life Sciences Nanjing University Nanjing 210008 China; ^4^ Departments of Plant Biology and of Crop Sciences University of Illinois at Urbana‐Champaign Urbana IL 61801 USA

**Keywords:** C_3_ photosynthesis, C_4_ photosynthesis, food security, future‐proofing agriculture, global change, nitrogen use efficiency, rising CO_2_, Rubisco

## Abstract

Global demand for food may rise by 60% mid‐century. A central challenge is to meet this need using less land in a changing climate. Nearly all crop carbon is assimilated through Rubisco, which is catalytically slow, reactive with oxygen, and a major component of leaf nitrogen. Developing more efficient forms of Rubisco, or engineering CO_2_ concentrating mechanisms into C_3_ crops to competitively repress oxygenation, are major endeavors, which could hugely increase photosynthetic productivity (≥ 60%). New technologies are bringing this closer, but improvements remain in the discovery phase and have not been reduced to practice. A simpler shorter‐term strategy that could fill this time gap, but with smaller productivity increases (*c*. 10%) is to increase leaf Rubisco content. This has been demonstrated in initial field trials, improving the productivity of C_3_ and C_4_ crops. Combining three‐dimensional leaf canopies with metabolic models infers that a 20% increase in Rubisco increases canopy photosynthesis by 14% in sugarcane (C_4_) and 9% in soybean (C_3_). This is consistent with observed productivity increases in rice, maize, sorghum and sugarcane. Upregulation of Rubisco is calculated not to require more nitrogen per unit yield and although achieved transgenically to date, might be achieved using gene editing to produce transgene‐free gain of function mutations or using breeding.

**Table:** 

	Contents	
	Summary	951
I.	Introduction	952
II.	How does Rubisco limit C_3_ photosynthesis?	953
III.	How does Rubisco limit C_4_ photosynthesis?	954
IV.	Strategies for increasing Rubisco carboxylation efficiency	956
V.	Simulating the impact of increased Rubisco on canopy photosynthesis in soybean (C_3_) and sugarcane (C_4_)	957
VI.	Approaches to increasing Rubisco content per leaf area	958
VII.	Will increased Rubisco content lower nitrogen use efficiency?	960
VIII.	Conclusions and future perspectives	962
	Acknowledgements	962
	References	962

## I. Introduction

**Fig. 1:**
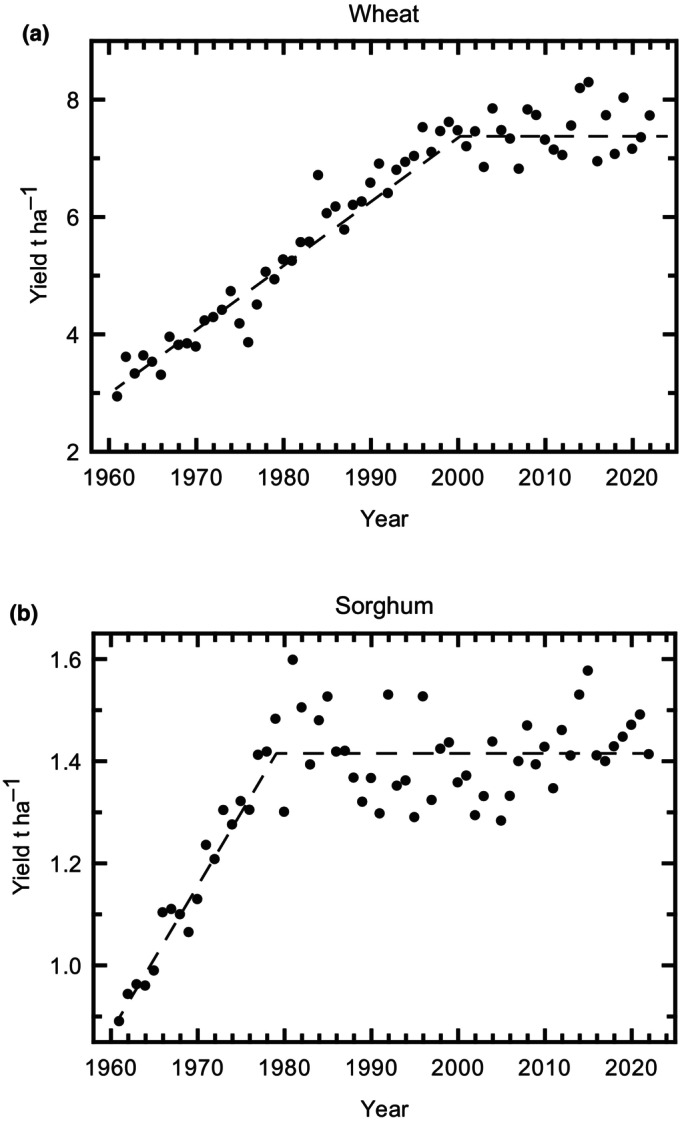
Average yields of wheat and sorghum over the last 60 yr. (a) Average wheat yields reported for France, Germany and the UK; and (b) average World sorghum yields. Yield refers to the harvested production per hectare for the area under cultivation. Official figures reported from 1961 to 2022 (FAOstat, 2024).

Remarkable increases in average yields of the major food crops have been achieved over the past 60 yr. However, projections of future demand based on rising population and increasing prosperity, particularly of the rapidly growing economies of East Asia, show that even if this rate of yield improvement is maintained, there is an ever‐widening gap between future demand and supply (Ray *et al*., 2013; van Dijk *et al*., 2021). In a summary of 57 global food security analyses, it was concluded that global food demand in 2050 will need to increase by 35–56% relative to production levels in 2010 (van Dijk *et al*., 2021). This is driven by three factors: (1) Global population is expected to grow from 7 billion in 2010 to 9.6 billion in 2050 (UN‐DES, 2024). (2) Production of meat and dairy per capita at a global level is showing a continuous increase, demanding more crop products per capita than vegetarian food stuffs. For example, global meat production in 2010 was 293 Mt and increased to 356 Mt in 2022, with this *c*. 1% per annum increase showing no sign of slowing (Ritchie *et al*., 2023). (3) Food waste increases as the proportion of the world population in urban centers grows, due to extended food chains and the greater affluence of residents of urban areas. Global food waste in 2019 was 962 Mt but is predicted to double by 2050 due to an additional 2.5 billion people living in urban areas (Forbes *et al*., 2021; Lopez Barrera & Hertel, 2021). That demand is outstripping production is partly reflected in the 735 million people estimated to have faced hunger in 2022, 122 million more than in 2019. This is compounded by civil unrest, which in itself can be precipitated by shortages (Soffiantini, 2020). Particularly affected by malnutrition were all regions of Africa, rural populations and women (FAO, 2024). Going forward this shortfall may be worsened by further factors, even in the absence of conflicts affecting global trade. The steady increases in yields seen over the last 60 yr may be stalling in some sectors (Ray *et al*., 2012; van Dijk *et al*., 2021). For example, France, Germany and the UK are the three largest producers of wheat in the EU27 + UK, with some of the highest yields per hectare in the world. Average yields across these three countries increased from *c*. 3 t ha^−1^ in 1960 to 7.5 t ha^−1^ in 2000, but in the last 22 yr there has been no discernable increase, despite significant breeding efforts (Fig. 1a). Similarly, average world yields of sorghum, a key crop for the drier tropical regions, has shown no improvement over the past 30 yr (Fig. 1b). The year‐on‐year increases before reaching these plateaus is largely attributed to the breeding of more productive cultivars, since fertilization levels in these countries were already high. The plateaus could in part result from environmental and management changes, but do show that breeding has not improved yield under these changed conditions. Projections of the impacts of climate change on yields has largely assumed steady increases in temperature and drought incidence (Wheeler & von Braun, 2013). Increasingly it has become apparent that extreme weather events, such as exceptional droughts, flooding and heat waves, are becoming more frequent. Taking such events into account suggests that up to an additional 36% of the world population could face hunger in 2050 (Cottrell *et al*., 2019; Hasegawa *et al*., 2021). Supply may be increased by expanding the agricultural footprint – causing more destruction of natural systems and acceleration of global change – or by increasing production on the land already in use. It is clearly critical and urgent that the latter is achieved by sustainably increasing resource and land use efficiency, but the former will be the reality if this goal is not met.

It is clear that innovations that can accelerate yield improvement and can be realized on a short timescale, as well as the longer term, are urgently needed to complement and integrate with ongoing crop breeding efforts. Photosynthesis falls well short of its theoretical potential, even in our most productive crops (Zhu *et al*., 2008). However, it was long considered that capacity to produce and fill the harvested part of the crop or sink was limiting, not photosynthesis (Richards, 2000; Borrás *et al*., 2004; Reynolds *et al*., 2005). This hypothesis can be tested experimentally via artificial elevation of [CO_2_], which, in the absence of other limitations, increases net photosynthesis. Many experiments exploring the impact of future elevated [CO_2_] have now shown that this artificial elevation of photosynthesis corresponds to large increases in crop yield, particularly in modern elite cultivars, suggesting that breeding has overcome sink limitation (Ainsworth & Long, 2020). Such findings have accelerated interest in genetically increasing crop photosynthetic efficiency by both breeding and bioengineering, utilizing the deep understanding of the photosynthetic process achieved over the last half‐century (Long *et al*., 2015).

All plants assimilate CO_2_ into carbohydrates via the single enzyme Ribulose‐1,5‐bisphosphate carboxylase/oxygenase (Rubisco). Two recent studies have shown that transgenic upregulation of Rubisco content in rice (C_3_) and sorghum (C_4_) resulted in significant increases in productivity in single‐site field trials (Yoon *et al*., 2020; Salesse‐Smith *et al*., 2024). These were achieved by either solely upregulating expression of a gene coding for the small subunit of Rubisco (*RbcS*) or upregulation of *RbcS* in combination with Rubisco Accumulation Factor 1 (*Raf1*). While these demonstrations were transgenic, they suggest the same might well be achieved by mutational editing of repressor elements in the promoter regions of these native genes, or perhaps by selection of lines with higher expression of Rubisco through marker assisted or genomic breeding (Theeuwen *et al*., 2022; Tang & Zhang, 2023). High‐throughput methods are now available to screen large amounts of germplasm and progeny of crosses. For example, hyperspectral scans coupled with regression analysis have been used to estimate variations in *V*_c,max_, the *in vivo* activity of Rubisco, in crops (Ainsworth *et al*., 2014; Silva‐Perez *et al*., 2018; Meacham‐Hensold *et al*., 2020). These approaches would likely avoid the lengthy delays and expense in deregulation currently required for transgenic plants. Thus, crop lines with upregulated Rubisco could become available to farmers relatively soon, providing an interim solution before larger yield increases are achieved through more complex pathway engineering approaches.

## II. How does Rubisco limit C_3_ photosynthesis?

Averaged over a broad range of herbaceous C_3_ plants, Rubisco was found to constitute 23.2 ± 5.5% of total leaf nitrogen (Onoda *et al*., 2017). This high content led in the past to the assumption that Rubisco was likely in excess of requirement for carboxylation and that this excess may serve as a nitrogen or ‘luxury’ reserve (Quick *et al*., 1991). However, early use of antisense mRNA showed that when grown under high‐light conditions, light‐saturated leaf CO_2_ uptake was linearly related to leaf Rubisco content (Hudson *et al*., 1992). The widely accepted and validated Farquhar–von Caemmerer–Berry (FvCB) model of steady‐state photosynthesis shows that the rate of leaf CO_2_ uptake (*A*) under any given set of conditions is limited by the lowest capacity of one of three processes: (1) the carboxylation of ribulose‐1,5‐bisphosphate (RuBP) by Rubisco (*V*_c,max_), (2) the rate of RuBP regeneration, which is typically determined by the rate of whole chain electron transport (*J*_max_), or (3) the capacity to export the products of C_3_ photosynthetic metabolism from the chloroplast as triose phosphates (*V*_TPU_), which can also control RuBP regeneration (Farquhar *et al*., 2001; Gregory *et al*., 2021). This model predicts that at low intercellular [CO_2_] (*C*_i_), light‐saturated *A* is limited by Rubisco activity (*V*_c,max_) and at higher *C*_i_ by RuBP regeneration (either *J*_max_ or *V*_TPU_). Therefore, if *C*_i_ is sufficient for *J*_max_ or *V*_TPU_ to be limiting, then in theory there would be no benefit in increasing Rubisco activity.

If all mesophyll cells within a leaf have equal capacities of *V*_c,max_ and *J*_max_, and the same [CO_2_] level at Rubisco, then the FvCB model predicts a sharp transition between Rubisco and RuBP limitation. However, heterogeneity will cause co‐limitation. Stomatal apertures across leaf surfaces show dynamic variation, resulting in patches where stomata are more open than others. This causes different *C*_i_ values under these patches, resulting in simultaneous limitation by Rubisco and RuBP in the same leaf (Farquhar *et al*., 2001). Gradients in light within the leaf may also cause some cells to be Rubisco activity limited while others are RuBP‐limited. This would extend the benefit of elevated *V*_c,max_ to a higher *C*_i_ than would be predicted when conditions and capacities are assumed spatially homogeneous in the leaf. Thus, any benefit from increased Rubisco activity is highly dependent on the point of transition, the sharpness of the transition, and the typical value of *C*_i_ during growth, referred to as the operating *C*_i_.

**Fig. 2:**
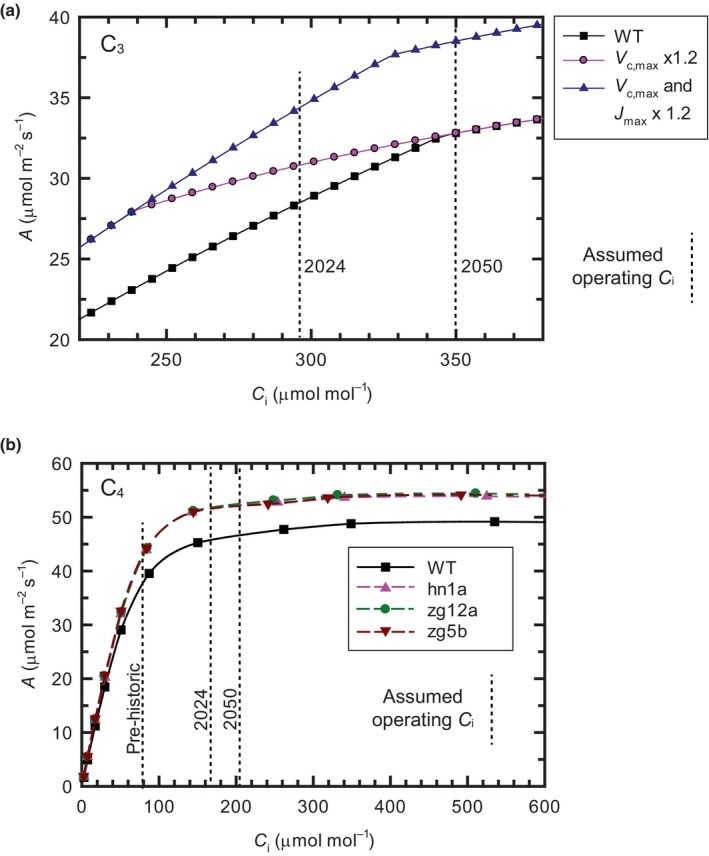
Effect of a 20% increase in Rubisco on *A* to *C*_i_ response curves in soybean (C_3_) and sorghum (C_4_). (a) ‘Wild‐type’ (WT) shows the modeled leaf CO_2_ uptake (*A*) vs intercellular CO_2_ concentration (*C*_i_) using the *V*_c,max_ and *J*_max_ reported for soybean accession PI561370 (Montes *et al*., 2022) as inputs to the FvCB equations (von Caemmerer, 2013). The effect of increasing Rubisco activity by 20% is represented by ‘*V*_c,max_ × 1.2’ and of increasing both Rubisco and capacity for RuBP regeneration by 20% by ‘*V*_c,max_ and *J*_max_ × 1.2’. Vertical dashed lines assume an operating *C*_i_, which is 0.7 of *C*_a_, where *C*_a_ in 2050 is estimated as *c*. 500 μmol mol^−1^. (b) ‘WT’ represents the measured mean *A* to *C*_i_ of sorghum line RTx430 compared to three independent lines of the same background, but with a transgenic upregulation of Rubisco of *c*. 25% (Salesse‐Smith *et al*., 2024). Vertical dashed lines represent the operating *C*_i_ of the past present and future, assuming a *C*_i_, which is 0.4 of *C*_a_.

In the absence of water stress, the ratio of *C*_i_ to atmospheric [CO_2_] (*C*_i_ / *C*_a_) is typically *c*. 0.7 in C_3_ plants (Ainsworth & Long, 2005). So at the current global atmospheric [CO_2_] of 424 μmol mol^−1^ (NOAA, 2024), the operating *C*_i_ would be *c*. 300 μmol mol^−1^. Average values of *V*_c,max_ and *J*_max_ for C_3_ annuals and perennials were obtained in a retrospective analysis of the responses of *A* to *C*_i_ for 109 C_3_ species (Wullschleger, 1993). Applying the FvCB equations (von Caemmerer, 2013) to these values shows that even in the current elevated [CO_2_] atmosphere, Rubisco activity would be limiting. However, in some of our major C_3_ crops, the transition between Rubisco and RuBP regeneration limitation appears to occur at lower *C*_i_, perhaps as a result of intensive selection. Applying the average *V*_c,max_ and *J*_max_ for soybean accession PI561370 (Montes *et al*., 2022) to the FvCB model predicts that the transition from Rubisco activity to RuBP‐limited assimilation would occur at a *C*_i_ of 364 μmol mol^−1^ (Fig. 2a), which, assuming *C*_i_ / *C*_a_ of 0.7, would correspond to an atmospheric [CO_2_] of *c*. 500 μmol mol^−1^. In this example, elevation of Rubisco activity by 20% would markedly increase light‐saturated leaf CO_2_ uptake (*A*) in the present atmosphere, but diminishing to zero increase by mid‐Century (Fig. 2a). However, there is considerable variation in average *V*_c,max_ and *J*_max_ across soybean germplasm (Montes *et al*., 2022). The transition was observed at a much lower *C*_i_ of 260 μmol mol^−1^ in soybean cultivar 93B15. The transition *C*_i_ rose slightly when the same cultivar was grown in elevated [CO_2_], suggesting some adaptive plasticity within genotypes (Bernacchi *et al*., 2005). Wheat with an adequate supply of nitrate showed a sharp transition from Rubisco activity to RuBP regeneration limitation at the operating *C*_i_ for the present atmosphere at 7 wk after anthesis, nearing the end of grain maturation. At 1 and 5 wk, however, the transition was not sharp suggesting co‐limitation by Rubisco and RuBP regeneration, which continued until a *C*_i_ of *c*. 400 μmol mol^−1^ (Evans, 1983). Rice by contrast did not show a transition to RuBP regeneration limitation until *C*_i_ > 500 μmol mol^−1^, and was therefore Rubisco limited at the operating *C*_i_ (Ziska & Teramura, 1992).

Thus, although there is variation in the transition point, many C_3_ crops and cultivars, especially rice and wheat but also soybean, are likely to benefit from increased Rubisco activity. Rising atmospheric [CO_2_] will shift limitation progressively away from Rubisco activity toward RuBP. However, droughts in agricultural regions are forecast to increase, which will decrease stomatal conductance such that *C*_i_ / *C*_a_ declines, bringing limitation back to Rubisco activity. In addition, in modern dense crop canopies, light fluctuations are frequent due to the movement of the sun, clouds and leaves. Leaf CO_2_ uptake recovers slowly on a sudden transfer of a leaf from shade to sun, lowering overall crop canopy assimilation. Analyses of the factors limiting this recovery shows Rubisco activity rather than electron transport as the key biochemical limitation in a range of C_3_ crops (Soleh *et al*., 2016; Acevedo‐Siaca *et al*., 2020; Taylor *et al*., 2022). Therefore, despite rising atmospheric [CO_2_], it can be anticipated that increasing Rubisco content or efficiency will be beneficial to C_3_ crops for some time to come.

## III. How does Rubisco limit C_4_ photosynthesis?

C_4_ crops, which include maize, sorghum and sugarcane, and the emerging bioenergy crops miscanthus, switchgrass and energycane, are some of the most efficient in terms of nitrogen and water use (Long, 1983; He *et al*., 2024). This is largely due to their ability to concentrate CO_2_ around Rubisco in bundle sheath (BS) chloroplasts (Furbank & Hatch, 1987). This CO_2_ concentrating mechanism (CCM) involves anatomical changes to the leaf, Kranz anatomy, which allows the partitioning of photosynthetic activities between two cell types, BS and mesophyll (M) cells. In addition, the biochemistry of C_4_ leaves is different from their C_3_ counterparts as two carboxylases are used to fix carbon sequentially. First, HCO_3_^−^ is fixed by phosphoenolpyruvate carboxylase (PEPC) in the M chloroplasts, and the resulting C_4_ dicarboxylates transported to the BS chloroplasts where they are decarboxylated and the resulting CO_2_ fixed by Rubisco. This higher [CO_2_] accelerates carboxylation and is sufficient to competitively suppress oxygenation and, in turn, photorespiration. As a result, C_4_ plants can achieve rates of photosynthesis equal to or higher than their C_3_ counterparts with less Rubisco, typically 20–30% of total leaf soluble protein, compared to *c*. 50% in C_3_ leaves (Feller *et al*., 2008).

Experimental studies and theory have shown that Rubisco is a major limiting step in C_4_ photosynthesis (von Caemmerer & Furbank, 2016; von Caemmerer, 2021). Transgenic knockdown of the Rubisco small subunit gene (*RbcS*) in the C_4_ plant *Flaveria bidentis* showed that Rubisco accounts for up to 70% of the control of light‐saturated photosynthetic rates at the thermal optimum and up to 99% at 6°C (Furbank *et al*., 1997; Kubien *et al*., 2003). In C_4_ plants, light‐saturated CO_2_ assimilation increases linearly with intercellular CO_2_ concentrations (*C*_i_) until reaching a plateau where assimilation rates remain constant with increasing *C*_i_ (von Caemmerer, 2021). The von Caemmerer model for C_4_ photosynthesis shows that PEPC activity (*V*_p,max_) is the main limitation to the initial slope of the *A* to *C*_i_ response, while Rubisco activity (*V*_c,max_) limits the plateau. This transition generally occurs around a *C*_i_ of 120 μmol mol^−1^ (Leakey *et al*., 2006). The *C*_i_ / *C*_a_ ratio for C_4_ plants is typically 0.4, corresponding to an operating *C*_i_ of 80 μmol mol^−1^ in the prehistoric atmospheric [CO_2_] of 220 μmol mol^−1^. Under these conditions, in which our crop ancestors evolved, the operating point of C_4_ photosynthesis resided predominantly on the PEPC limited portion of the *A* to *C*_i_ response (prehistoric, Fig. 2b). Increases in atmospheric CO_2_ of the last 50 yr have pushed the operating point into the Rubisco limited range (Pignon & Long, 2020) (Fig. 2b). Unlike in C_3_ plants, Free‐Air Concentration Enrichment (FACE) studies have shown that under nonstress conditions, higher CO_2_ concentrations do not increase photosynthetic efficiency and productivity of C_4_ plants, indicating C_4_ photosynthesis is CO_2_ saturated (Leakey *et al*., 2006; de Souza *et al*., 2013; Ainsworth & Long, 2020). Essentially, this means that CO_2_ assimilation rates are expected to become increasingly Rubisco limited in C_4_ plants as atmospheric CO_2_ levels continue to rise. Rubisco content has also been strongly associated with cold‐temperature tolerance of C_4_ plants (Kubien *et al*., 2003; Wang *et al*., 2008; Long & Spence, 2013). With increasing variability around average temperatures, cold stress is likely to remain an issue for early‐ and late‐season C_4_ crops in temperate climates. Thus, increasing Rubisco content could be a valuable strategy to improve carbon uptake in C_4_ crops, especially during cold spells, helping to future‐proof these crops.

So far, limitations to steady‐state light‐saturated C_4_ photosynthesis have been considered. However, most growth environments outside of controlled environment agriculture are dynamic. As in C_3_ crops, modern C_4_ crop canopies are dense and comprised of several layers of many leaves that experience frequent shade to sun transitions (Long *et al*., 2022). A mechanistic model parameterized to specific cultivars of maize, sorghum and sugarcane inferred Rubisco activation as the major cause of the lag in increase in CO_2_ assimilation on transfer to sun (Wang *et al*., 2021). If correct, then during induction, more CO_2_ would be pumped into the BS than can be used by Rubisco, resulting in a higher CO_2_ concentration than at steady‐state and, in turn, more diffusive CO_2_ leakage back to the mesophyll (BS leakiness; ɸ). If the limitation was within the mesophyll or related to decarboxylation rather than Rubisco, BS leakiness would be less. A new method coupling gas exchange with carbon isotope discrimination measurements allows for continuous monitoring of BS leakiness under dynamic light conditions to test this hypothesis. Experimental analyses showed that BS leakiness during induction in maize and sorghum was almost double that at steady‐state, confirming the limitation at Rubisco (Wang *et al*., 2022). If the amount of active Rubisco at any point during induction is proportional to the total amount of Rubisco, then increasing leaf Rubisco content would also improve photosynthetic efficiency in fluctuating light. Transgenic elevation of Rubisco by 13–25% in sorghum increased the speed of induction on shade to sun transitions (Salesse‐Smith *et al*., 2024). However, some studies reported decreased Rubisco activation when Rubisco content was increased (Suzuki *et al*., 2007; Ishikawa *et al*., 2011; Salesse‐Smith *et al*., 2018). Though these lines still had increased levels of total active Rubisco, a greater advantage would likely be obtained if Rubisco and Rubisco activase (*Rca*) are upregulated in tandem (Wang *et al*., 2021).

## IV. Strategies for increasing Rubisco carboxylation efficiency

Form I Rubisco is a hexadecamer made up of 8 large subunits (LSU) and 8 small subunits (SSU). This form is found in a number of bacterial lineages including cyanobacteria, and all photosynthetic eukaryotes (Prywes *et al*., 2023). In plants and algae, LSU is encoded by the chloroplast gene *rbcL* and SSU is encoded by the nuclear gene family *RbcS*. The Rubisco holoenzyme (L_8_S_8_) active site is located at the interface between two LSUs and has strong structural and sequence identity between species (Parry *et al*., 2003). The SSU has more sequence diversity between species and is not involved in active site formation, though it can influence catalytic activity (Lin *et al*., 2020; Mao *et al*., 2023). Form I Rubisco has a complex biogenesis pathway best described in cyanobacteria (Hauser *et al*., 2015). In short, Form I Rubisco requires the assistance of 4 assembly chaperones, 1 folding chaperone and 1 catalytic metabolic repair chaperone protein (Hayer‐Hartl, 2017; Gionfriddo *et al*., 2023). Enhancing Rubisco carboxylation rates has long been a target for improving photosynthesis, growth and yield under current and future elevated CO_2_ concentrations. This is in part due to its relatively slow catalytic rate and reactivity with oxygen. There have been numerous efforts to improve Rubisco kinetic properties or alternatively increase the CO_2_ concentration around Rubisco to improve its efficiency, which will be discussed here.

The kinetic traits of Rubisco, such as its CO_2_ fixing speed (*k*_cat_), carboxylation efficiency and CO_2_ specificity (*S*_c/o_), have been targeted for improvement through mutagenesis, subunit swapping, screening natural diversity and directed evolution. To date, mutagenesis studies have not been successful in generating Rubiscos with major kinetic improvements. In fact, mutation of the conserved residues surrounding the LSU active site has generally led to decreases in *k*_cat_ and *S*_c/o_ (Prywes *et al*., 2023). In instances where improvements from point mutations have been reported, they are generally quite minor and result in a trade‐off between kinetic traits. In fact, improved *k*_cat_ often results in a reduction in specificity or the inverse (Flamholz *et al*., 2019). However, generating Rubiscos with kinetic improvements is within reach as new systems for introducing stable point mutations into *rbcL* are being developed, which will allow more high‐throughput assessment of mutant Rubiscos *in planta* (Lin *et al*., 2021). Nevertheless, modifications of *rbcL* in crops will be limited to species amenable to chloroplast transformation, which remain few. At present chloroplast transformation remains a large bottleneck in monocots (Rascón‐Cruz *et al*., 2021), a group that includes rice, wheat, sorghum and maize, some of the most important crop species.

Enzymes with improved kinetic traits have been identified in nature by screening Rubisco from different species, with some of the most efficient forms found in red algae (Orr *et al*., 2016; Oh *et al*., 2023; Y. Zhou *et al*., 2023). Much unexplored natural diversity also exists, particularly in bacterial Rubiscos, indicating a potential to replace native crop Rubiscos with those with improved catalytic capacity in order to improve crop productivity (De Pins *et al*., 2024; Prywes *et al*., 2024). The major challenge here is that there are incompatibilities between host plant chaperones and heterologous Rubisco subunits from algae or bacteria, which prevents successful assembly of these improved Rubiscos into plants (Parry *et al*., 2013). As such, plant growth has not been improved by any subunit swapping studies, often due to limitations in holoenzyme assembly (Sharwood *et al*., 2007; Lin & Hanson, 2018). Complicating this further, *rbcL* was recently shown to be among the top 1% of slowest evolving genes on Earth (Bouvier *et al*., 2024). Taken together, this has led to a growing interest in using directed evolution – rapid and iterative laboratory selection of proteins with improved biological fitness – to increase Rubisco catalysis (Gionfriddo *et al*., 2024). The same challenges with regards to chloroplast transformation, heterologous replacement of Rubisco and chaperone compatibility will persist, however. Further mechanistic understanding of the Rubisco biogenesis pathway and development of chloroplast transformation systems in monocots will help to address these challenges. A key question will be whether the trade‐off between kinetic parameters that has typically been seen in nature continues in laboratory evolved Rubisco.

In contrast to the identification or development of more efficient forms of the enzyme, introducing CCMs into C_3_ crops also has the potential for significant gains in plant productivity (von Caemmerer *et al*., 2012; McGrath & Long, 2014; Fei *et al*., 2022). Green algae and cyanobacteria use pyrenoid‐based and carboxysome‐based biophysical CCMs, respectively, to concentrate CO_2_ around Rubisco (Price & Badger, 1991; Morita *et al*., 1999). Massive advances in unravelling the combination of genes coding for these structures is bringing the prospective of engineering these into plants closer (Long *et al*., 2018; Fei *et al*., 2022; Förster *et al*., 2023; He *et al*., 2023; Nguyen *et al*., 2023; Rottet *et al*., 2024). Given the large increase in photosynthetic efficiency, potentially 60%, and feasibility of these approaches, it would appear imperative that these are pursued and translated into C_3_ crops. Today, plants use the biochemical CCMs of C_4_ photosynthesis and Crassulacean acid metabolism to elevate the CO_2_ concentration at Rubisco. Considerable progress has been made toward engineering a C_4_ rice (Furbank *et al*., 2023). An even longer‐term solution may be avoiding Rubisco altogether by engineering alternative synthetic carbon fixing cycles into crops, but integrating these pathways with plant metabolism to replace the Calvin‐Benson‐Bassham cycle and its integration into multiple metabolic pathways will be a great bioengineering challenge (Correa *et al*., 2023; Schulz‐Mirbach *et al*., 2024).

In summary, although substantial research breakthroughs have been made, such as the reconstitution of plant Rubisco *in vitro* and assembly of carboxysomes in plant chloroplasts (Aigner *et al*., 2017; Long *et al*., 2018), improvements in photosynthesis and yield have not yet been achieved by improved Rubisco variants or bioengineered CCMs. However, there would appear no barrier to these being achieved with more time and support. A more detailed understanding of plant Rubisco biogenesis is still needed to introduce better Rubiscos identified through mutagenesis, natural variation or directed evolution into crops. In particular, a better understanding of the complementarity requirements between Rubisco subunits and their assembly and metabolic repair chaperones in needed (Gunn *et al*., 2020). The strategies discussed here have massive potential for crop improvement; however, we are still many years away from attaining the gains currently required. In the meantime, increasing Rubisco protein content has resulted in increased photosynthesis and crop productivity, and could be a simple strategy to attain moderate gains on a shorter timescale, given that it has been reduced to practice in both a C_3_ and a C_4_ food crop. This is discussed in the following section. Table 1 summarizes the primary approaches for developing more efficient Rubisco forms, including the speculated timescales, benefits and challenges associated with each.

**Table 1:** Summary of the key approaches for increasing Rubisco carboxylation efficiency, and the benefits and challenges associated with each.

Goal		Time scale	Regulation friendly	Needs plastid transformation	Crop net carbon gain		Minimum number of genes to manipulate
					C_3_	C_4_	
**Improve Rubisco Kinetics by 20%**							
A	Mutagenesis and Editing	Medium	Yes	*rbcL* – Yes, *RbcS* – No	7–27%^a^	15%^e^	1–2
B	Screening natural diversity for replacement of native crop Rubiscos	Long	No	*rbcL* – Yes, *RbcS* – No	7–27%^a^	15%^e^	1–2
C	Directed evolution	Medium	No	*rbcL* – Yes, *RbcS* – No	7–27%^a^	15%^e^	1–2
**Introduce CCM into C**_**3**_ **crops**							
A	Convert C_3_ plants to C_4_	Long	No	No	30%^b^	n/a	> 20^f^
B	Add algal pyrenoid system	Long	No	Ideally	60%^i^	0%	> 5^g^
C	Add cyanobacterial carboxysome system	Long	No	Ideally	60%^c^	0%	> 4^h^
Increase Rubisco protein content		Short	Yes	No	10%^d^	15%^e^	1–3

Short represents a 1–5 yr timescale, medium 5–10 yr and long > 10 yr. ‘Ideally’ means the manipulation is possible without plastid transformation but this would introduce additional complications. Speculated increases in carbon assimilation are from modeled estimates and untested aside from increased Rubisco content (Salesse‐Smith *et al*., 2018, 2024; Yoon *et al*., 2020). Table shows approximate minimum number of genes required to introduce each CCM into C_3_ crops as the exact numbers are not yet known.

^a^ Gionfriddo *et al*. (2024).

^b^ von Caemmerer *et al*. (2012).

^c^ McGrath & Long (2014).

^d^ See Fig. 4.

^e^ See Fig. 3.

^f^ Ermakova *et al*. (2020).

^g^ Barrett *et al*. (2021).

^h^ Long *et al*. (2018).

^i^ Fei *et al*. (2022).

## V. Simulating the impact of increased Rubisco on canopy photosynthesis in soybean (C_3_ ) and sugarcane (C_4_ )

The previous sections have considered limitations at the level of light‐saturated single leaves and the role of Rubisco in limiting CO_2_ uptake. Relevant to crop production is the total CO_2_ uptake of the canopy of leaves over the course of the day. Here many leaves will be shaded by other leaves or in low light at the beginning and end of the day. In shade, CO_2_ uptake is limited by the supply of reductant and ATP, rather than carbon metabolism. Therefore, the benefit of increasing Rubisco will be less in a crop canopy in the field than for a single leaf under light‐saturating conditions, but by how much less? Prediction is complicated by sunflecking. That is, as the sun angle changes over the course of the day, rays penetrate to lower canopy leaves giving them periods of light‐saturation, a process termed sunflecking. Previously, we have combined the 3D canopy structures of sugarcane (C_4_) and soybean (C_3_) with a ray tracing algorithm and then used the dynamic lighting of all leaves in the canopy to predict carbon assimilation based on steady‐state models of photosynthetic CO_2_ assimilation (Wang *et al*., 2017, 2020). These models are re‐used here to predict the benefit of a 20% upregulation of Rubisco activity (*V*_c,max_).

**Fig. 3:**
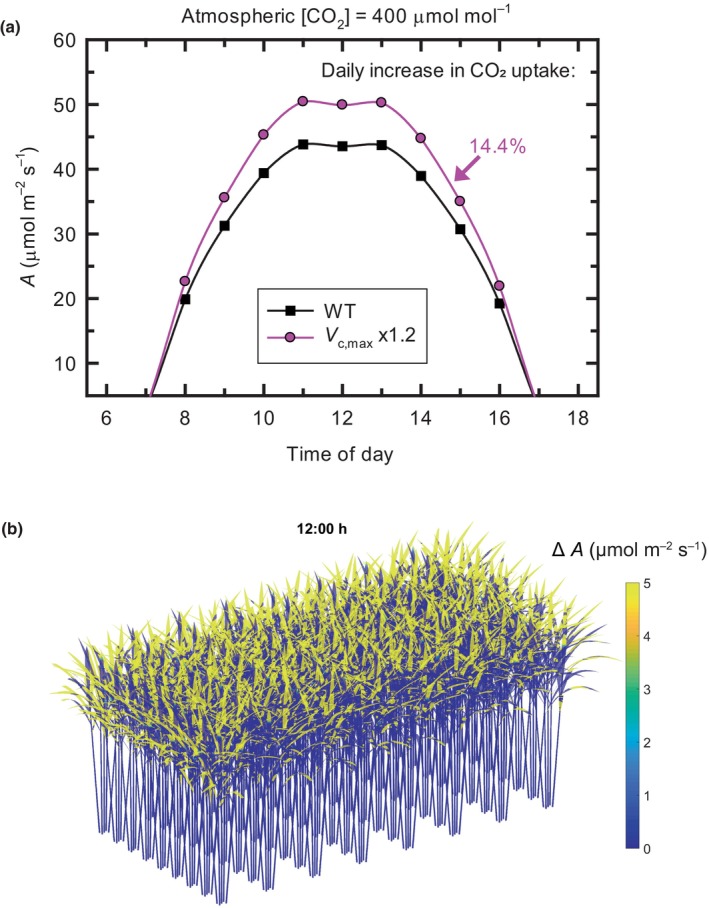
Enhancement of canopy CO_2_ assimilation rates in sugarcane from a 20% increase in Rubisco. (a) ‘Wild‐type’ (WT) shows the modeled diurnal crop photosynthetic CO_2_ uptake as described previously for a mature sugarcane canopy in the present atmosphere (Wang *et al*., 2017). The effect of increasing Rubisco activity by 20% is represented by ‘*V*_c,max_ × 1.2’. The percent increase in CO_2_ uptake is integrated over the day relative to ‘WT’. (b) The enhancement of leaf net CO_2_ assimilation from additional 20% Rubisco (Δ*A*) in a modeled sugarcane canopy (*Saccharum officinarum*, RB86‐7515) at 12:00 h on 20 July, Sao Paulo, Brazil. The row spacing of the canopy is 100 cm. Leaf area index is 5.8. Colors indicate the spatial heterogeneity of the change in *A* throughout the canopy at 12:00 h.

**Fig. 4:**
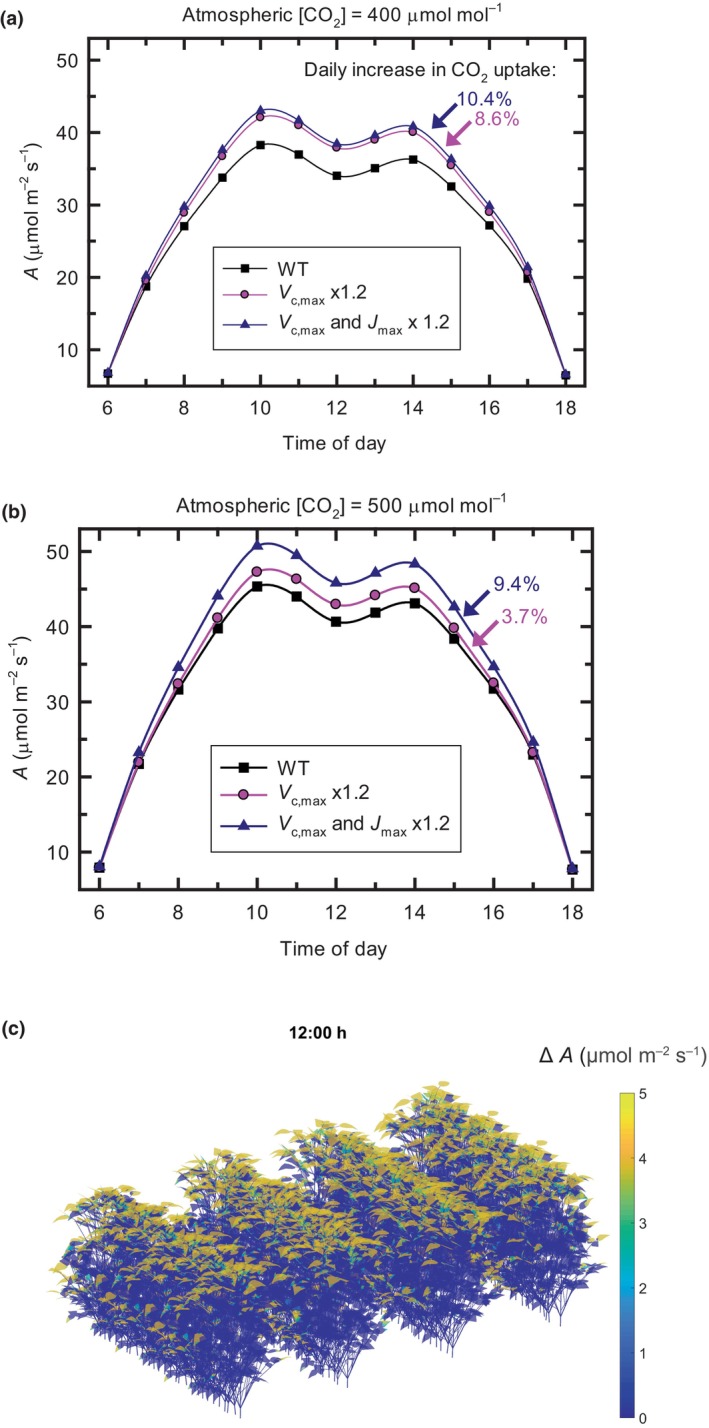
Enhancement of canopy CO_2_ assimilation rates in soybean from a 20% increase in Rubisco. (a) ‘Wild type’ (WT) shows the modeled diurnal crop photosynthetic CO_2_ uptake as described previously for a mature soybean canopy in the present atmosphere (Wang *et al*., 2020). The effect of increasing Rubisco activity by 20% is represented by *V*_c,max_ × 1.2 and of increasing both Rubisco and capacity for RuBP regeneration by *V*_c,max_ and *J*_max_ × 1.2. The percent increase in CO_2_ uptake integrated over the day relative to ‘WT’. (b) As for (a) but at a future atmospheric [CO_2_] of 500 μmol mol^−1^. (c) The enhancement of leaf net CO_2_ assimilation from additional 20% Rubisco (Δ*A*) in a modeled soybean canopy (*Glycine max* L. Merr., LD11‐2170) at 12:00 h on 18 August, Champaign, IL, USA. The atmospheric [CO_2_] is 400 μmol mol^−1^, the row spacing of the canopy is 76 cm, and plant spacing within the rows 5 cm. Leaf area index is 5.9. Colors indicate the spatial heterogeneity of the change in *A* throughout the canopy at 12:00 h.

In C_4_ sugarcane, this 20% increase in Rubisco activity resulted in a 14.4% increase in total CO_2_ uptake by the canopy over the course of the day (Fig. 3). This was independent of whether the simulation was at an atmospheric CO_2_ mole fraction of 400 μmol mol^−1^, approximating the present atmosphere, or 500 μmol mol^−1^, as anticipated for mid‐century. For soybean, a 20% increase in *V*_c,max_ was predicted to increase crop CO_2_ assimilation by 8.6% in the current atmosphere, but only 3.7% at an atmospheric [CO_2_] of 500 μmol mol^−1^, suggesting that any gain would diminish over the coming decades (Fig. 4a,b). However, in C_3_ crops, the benefit of increasing *V*_c,max_ will be heavily dependent on the rate of RuBP regeneration. This has been successfully increased in soybean and tobacco by upregulation of enzymes involved in regeneration of RuBP within the Calvin‐Benson‐Bassham cycle and components of the chloroplast electron transport chain (Köhler *et al*., 2017; Lopez‐Calcagno *et al*., 2020; Raines, 2022). When *V*_c,max_ and whole chain electron transport (*J*_max_), assumed to govern RuBP regeneration, were both increased by 20%, then canopy assimilation was increased by 10.4% at an atmospheric [CO_2_] of 400 μmol mol^−1^ and an only slightly lower 9.4% at a future 500 μmol mol^−1^ (Fig. 4; Supporting Information Video S1). Simultaneous upregulation of *J*_max_ therefore appears a means to future‐proof the benefit of upregulating Rubisco activity in C_3_ crops. These simulations were for mature crops with a high‐leaf area index (LAI). It could be anticipated that greater gains would be achieved at earlier growth stages, when a higher proportion of the canopy will be light‐saturated. Given that Rubisco is an exceptionally large protein of *c*. 550 kDa, there will be significant ATP costs in synthesizing an additional 20%, ranging from synthesis of the constituent amino acids to maturation of the protein. Analysis of these costs shows, using rice as an example, that the extra photosynthesis resulting from an additional 20% of Rubisco would repay the metabolic cost in just under 1 d (Table S1). This does not take account of the fact that Rubisco is one of the first proteins to be mobilized during seed/grain filling, such that a significant portion of its carbon and nitrogen will contribute to the harvested product (Nie *et al*., 1995; Mu *et al*., 2018).

## VI. Approaches to increasing Rubisco content per leaf area

### 1. Transgenic approaches

As discussed, a potentially shorter‐term approach is to increase Rubisco abundance within chloroplasts in order to increase Rubisco carboxylation rates and hence CO_2_ assimilation rates. In fact, both controlled environment and recent replicated single‐site field trials in C_3_ and C_4_ crops have shown that simply increasing the amount of Rubisco results in significant increases in productivity. In rice, overexpression of the native *RbcS* gene was shown to increase Rubisco content by 30% on a leaf area basis (Suzuki *et al*., 2007). Only upregulation of the *RbcS* gene was needed here to achieve increased Rubisco levels, presumably because *rbcL* translation is regulated by the supply of SSU protein (Wostrikoff & Stern, 2007; Wietrzynski *et al*., 2021). Growth chamber characterization of these rice plants overexpressing *RbcS* showed 15% increases in total dry biomass production when grown at 400 μmol mol^−1^ CO_2_ (Sudo *et al*., 2014). These plants were then grown in an experimental paddy field where they showed yield increases of 20–28% over a 4 yr period when sufficient N was applied (Yoon *et al*., 2020).

Transgenic overproduction of Rubisco has also been shown to improve productivity in some of the most economically important C_4_ crops: maize, sorghum and sugarcane (Salesse‐Smith *et al*., 2018, 2024). In C_4_ plants, *RbcS* and the assembly chaperone Rubisco Accumulation Factor 1 (*Raf1*) were overexpressed in tandem, as prior work in maize showed overexpression of *RbcS* alone or together with *rbcL* did not increase total Rubisco levels (Wostrikoff & Stern, 2007). In maize, upregulation of native *RbcS* and *Raf1* resulted in a 30% increase in Rubisco content, 15% increase in light‐saturated CO_2_ assimilation rates and *c*. 30% increase in plant biomass under optimal and chilling conditions in controlled environments (Salesse‐Smith *et al*., 2018, 2020). Upregulation of the same maize genes in grain sorghum cv. RTx430 produced up to a 25% increase in Rubisco accumulation, which resulted in similar increases in light‐saturated CO_2_ assimilation rates and ultimately a 16% increase in vegetative biomass in unfertilized replicated plots in the field (Salesse‐Smith *et al*., 2024). In addition, assimilation of CO_2_ was faster and greater during the transition from dark to light, an important trait for maintaining photosynthetic efficiency and productivity under fluctuating light conditions experienced in crop canopies (Wang *et al*., 2021; Long *et al*., 2022). In sugarcane, upregulation of sorghum *RbcS* and *Raf1* increased Rubisco on a leaf area basis up to 90%, resulting in a 31–81% increase in leaf and stem dry biomass in 2 month old plants grown under glasshouse conditions. Further testing will be required to determine whether these improvements will translate into parallel economic yield improvements in the field. Another open question is whether stacking upregulation of the other assembly factors required for Rubisco assembly *in planta* leads to additive increases in plant performance (Hotto *et al*., 2021).

Studies increasing Rubisco content in rice and maize have also reported corresponding decreases in Rubisco activation (Suzuki *et al*., 2007; Ishikawa *et al*., 2011; Salesse‐Smith *et al*., 2018). Furthermore, overexpression of Rubisco activase (*Rca*) in rice has been shown to be negatively correlated with Rubisco content (Fukayama *et al*., 2012, 2018). This reveals a potential drawback to upregulating Rubisco content, as this may introduce a RCA limitation resulting from a downstream feedback response. However, upregulating *Rca* in tandem with *RbcS* may be sufficient to override this feedback mechanism, producing more RCA protein to activate the extra Rubisco and alleviating its limitation to further increases in photosynthetic rates. This may be of particular importance in dynamic light environments and under rising temperatures (Scafaro *et al*., 2016; Qu *et al*., 2021; Amaral *et al*., 2024). In C_4_ crops, Pyruvate Pi dikinase (PPDK) shares metabolic control with Rubisco under current and future atmospheric [CO_2_] (Furbank *et al*., 1997), and increasing Rubisco would increase PPDK control. Mechanistic modeling suggests the greatest advantage would result from co‐upregulation of Rubisco and PPDK, together with RCA and the PPDK regulatory protein (PDRP) (Wang *et al*., 2021).

### 2. Gene editing approaches

Upregulation of Rubisco has so far been achieved by either transgenic addition of extra copies of native *RbcS* and/or *Raf1* genes, or transgenic addition of copies from closely related species. While this has the advantage of having already been shown to be successful in some crops, entry to seed systems will require extensive testing over several years to meet regulatory requirements. An alternative approach for upregulating Rubisco is to use DNA editing (CRISPR/Cas) to produce gain of function mutations by targeting plant promoters or upstream open reading frames (uORFs).

Promoters are regions of DNA found upstream of a gene where RNA polymerase and transcription factors bind to initiate transcription. One way to increase endogenous gene expression is to fuse inactive Cas proteins with transcriptional activators (Ming *et al*., 2020; Liu *et al*., 2022). This strategy has been shown to increase expression of a target gene by 10‐fold in stably transformed rice plants (Liu *et al*., 2022). Alternatively, gene expression can be increased using epigenetic modifications by fusing inactive Cas proteins to demethylases to alter DNA methylation levels in the promoter (Tang & Zhang, 2023). However, at present these gain of function edits require the plants to keep their CRISPR constructs and are therefore are considered transgenic for regulatory purposes. Promoter bashing – using CRISPR/Cas to create short deletions in the promoter region of a target gene – is another method to modulate transcription levels. This strategy has been successful in producing transgene‐free gain of function mutations resulting in crop improvements, but phenotyping the edited plants can be quite laborious and promoter bashing often does not increase expression of the edited gene. More efficient promoter editing systems are now being developed which could overcome this limitation (J. P. Zhou *et al*., 2023).

A second and perhaps more promising approach for upregulating Rubisco is to identify repressive uORFS in native *RbcS, Raf1* and *Rca* genes and mutate them using DNA editing. Upstream open reading frames are regulatory elements located in the 5′ untranslated regions (UTR) of messenger RNA and often suppress downstream translation of the primary uORFs. In recent years, screening methods have been developed using dual‐fluorescence or dual‐luciferase reporter systems to fine‐tune gene expression by editing endogenous uORFs using CRISPR (Si *et al*., 2020; Wu *et al*., 2022). These methods can be used to identify repressive uORFs in the 5′UTRs of genes of interest, which can then be mutated using CRISPR to upregulate endogenous protein expression. This approach has the advantage of producing transgene‐free gain of function mutations in plants, which in many jurisdictions would likely be allowed without the deregulation required of transgenic crops. Editing uORFs has successfully produced transgene‐free strawberries with increased sugar content, and lettuce plants with increased ascorbate acid content, demonstrating the potential of this method for crop improvement (Zhang *et al*., 2018; Xing *et al*., 2020).

### 3. Breeding approaches

Both of the above approaches would require integration with ongoing breeding efforts, including identifying germplasm that would provide the best backgrounds for maximizing trait benefits. It should not be overlooked that Rubisco content might be upregulated through targeted breeding such as marker assisted approaches or genomic selection. Methods for high‐throughput screens of Rubisco activity *in vitro* and *in vivo* are now available facilitating screening across crop germplasm (Sales *et al*., 2020; Montes *et al*., 2022). A wheat study of 64 cultivars showed that the Rubisco content of flag leaves varied *c*. 1.5‐fold (Carmo‐Silva *et al*., 2017), while an approximately two‐fold variation in *in vivo* Rubisco activity (*V*_c,max_) was found in modern North American soybean germplasm (Montes *et al*., 2022), suggesting an opportunity to test the combination of increased Rubisco content and activity with other yield traits. However, more large‐scale screens of Rubisco activity similar to the ones mentioned are urgently needed across additional crop germplasm.

## VII. Will increased Rubisco content lower nitrogen use efficiency?

At *c*. 20% of total leaf nitrogen, Rubisco accounts for more N than any other single leaf protein in C_3_ crops, and 8% in C_4_, making it by far the most abundant individual protein on the planet (Makino *et al*., 2003; Evans & Clarke, 2018; Bar‐On & Milo, 2019). These C_3_ vs C_4_ differences are attributed to the high [CO_2_] in which Rubisco operates within C_4_ leaves. Fig. 2 shows that under the low [CO_2_] in which the crop ancestors evolved Rubisco was the major limitation to photosynthesis in C_3_, but not C_4_ species, hence there would have been strong selective pressure for higher Rubisco contents only in C_3_ species. Increasing the amount of Rubisco to gain more activity will be less efficient in nitrogen use than developing catalytically more efficient forms. So, would increasing the Rubisco content from its already high levels worsen nitrogen use efficiency (NUE) at a time when there is considerable pressure and need to decrease nitrogen use in agriculture (Robertson & Vitousek, 2009)? NUE can be defined as the ratio between nitrogen in the harvested grain and the nitrogen in the entire crop, where a higher value of this dimensionless number indicates that more of the nitrogen used by the crop is available in the final product (Congreves *et al*., 2021). Thus, the influence of increased Rubisco content on NUE hinges on whether the nitrogen cost of extra Rubisco is outweighed by additional nitrogen contained in the larger yield. Rising atmospheric [CO_2_] tends to decrease seed N content, yet breeders have resources to counter this due to a wide variation (> twofold) in N content within major crop germplasm (Ainsworth & Long, 2020). The increase in yield resulting from a 20% increase in Rubisco content will demand more N if grain N content and quality is maintained. Will this increase in N demand for additional yield, be more or less than that required to increase leaf Rubisco content by 20%?

The N content of young maize leaves was reported as 90 mmol m^−2^, of which 8.5% was Rubisco (Makino *et al*., 2003). If a leaf area index (LAI) of six at grain filling is assumed and that this concentration is maintained to maturity, the total crop N would be 75.6 kg ha^−1^ and the contribution of Rubisco to crop N content would be 6.4 kg ha^−1^, so increasing the content by 20% would require a further 1.3 kg [N] ha^−1^ (Table 2). Fig. 4 predicts that a 20% increase in Rubisco activity would increase canopy carbon gain at this stage by 14.4%. Average maize yield in the USA in 2022 was 10.9 t ha^−1^ (FAO, 2024) and if all of the canopy carbon gain predicted translated into yield, that would increase yield by 1.6 t ha^−1^. To maintain a grain protein content of 10% (Shewry, 2007), this would require an additional 25.11 kg [N] ha^−1^ (Table 2). In the baseline situation, NUE is estimated to be 0.70, while including the additional N costs (in Rubisco and grain) and N return alters estimated NUE to 0.72 (Table 2). The N content of young rice leaves was reported as 124 mmol m^−2^, of which 27% was Rubisco (Makino *et al*., 2003). Applying the same reasoning in the case of rice, and despite its much higher Rubisco content and lower predicted yield gain, the baseline NUE is 0.43 while NUE with 20% more Rubisco is 0.44 (Table 2). In both cases, increasing Rubisco content would not lower nitrogen use efficiency. These simple calculations likely over‐estimate the cost of Rubisco, since they are based on the contents of young leaves and Rubisco content typically declines with depth into mature canopies (Townsend *et al*., 2017). They also neglect catabolism of Rubisco during leaf senescence, which is a major source of nitrogen for the developing grain of crops (Hirel & Gallais, 2006). Since the additional N in the grain exceeds the additional N in Rubisco for both crops, it may be fully or partially retranslocated to the grain, further reducing the overall N required by the crops.

**Table 2:** Nitrogen cost of 20% more Rubisco relative to the requirement for additional grain yield and its influence on nitrogen use efficiency.

	Units	Maize	Rice
**Nitrogen cost of extra Rubisco**			
Leaf area index (LAI)	m^2^ leaf (m^2^ ground)^−1^	6	6
Leaf N content	mmol [N] (m^2^ leaf)^−1^	90	124
Crop total N	kg [N] ha^−1^	75.6	104.2
Rubisco N fraction	%	8.5	27
Leaf Rubisco N	mg [N] (m^2^ leaf)^−1^	107.1	468.7
Crop Rubisco N	kg [N] ha^−1^	6.4	28.12
Crop additional Rubisco N	kg [N] ha^−1^	1.3	5.62
**Nitrogen cost of extra yield**			
Crop CO_2_ gain	%	14.4	8.6
Average yield	t ha^−1^	10.9 (USA, 2022)	7.1 (China, 2022)
Yield gain	t ha^−1^	1.6	0.61
Protein in grain	%	10	7
N in yield	kg [N] ha^−1^	174.4	79.52
Additional harvest N	kg [N] ha^−1^	25.11	6.84
N cost ratio	Dimensionless	19.5	1.2
**Nitrogen use efficiency**			
Baseline NUE	Dimensionless	0.70	0.43
Extra Rubisco NUE	Dimensionless	0.72	0.44

‘LAI’, Crop leaf area per unit ground area the start of grain fill; ‘Leaf N content’, Molar amount of N per unit leaf area; ‘Crop total N’, Mass of N in crop leaves per unit ground area, calculated as the product of ‘LAI,’ ‘Leaf N content,’ and the molar mass of N (14 g mol^−1^); ‘Rubisco N fraction’, Fraction of leaf N attributable to Rubisco; ‘Leaf Rubisco N’, Mass of N attributable to Rubisco per unit leaf area, calculated as the product of ‘LAI,’ ‘Leaf N Content,’ ‘Rubisco N fraction,’ and the molar mass of N; ‘Crop Rubisco N’, Mass of N in crop leaves attributable to Rubisco per unit ground area, calculated as the product of ‘LAI’ and ‘Leaf Rubisco N’; ‘Crop Additional Rubisco N’, Mass of N per unit ground area of an additional 20% Rubisco; ‘Crop CO_2_ gain’, Relative increase in crop CO_2_ uptake with a 20% increase in Rubisco (Fig. 3); ‘Average yield’, Average country yields for 2022 (FAOstat, 2024); ‘Yield gain’, Additional yield that would result if all additional crop CO_2_ uptake is translated into an equal increase in yield; ‘Protein in grain’, Fraction of grain mass that is protein (Shewry, 2007); ‘N in yield’, Mass of N harvested per unit ground area, assuming that N constitutes 16% of the mass of grain protein, calculated from the product ‘Average yield’ and ‘Protein in grain’; ‘Additional harvest N’, Mass of additional N harvested per unit ground area due to a 20% increase in Rubisco, calculated from the product of ‘Crop CO_2_ gain’ and ‘N in yield’; ‘N cost ratio’, ‘Additional harvest N’ divided by ‘Crop additional Rubisco’. A ratio greater than one indicates that the increase in N demand for additional yield is more than that required to increase leaf Rubisco content by 20%; ‘Baseline NUE’, the ratio of harvested N (‘N in yield’) to N inputs (assumed to be the total N in leaves and grain, the sum of ‘N in yield’ and ‘Crop total N’) for a typical crop; ‘Extra Rubisco NUE’, the ratio of harvested N (the sum of ‘N in yield’ and ‘Additional harvest N’) to N inputs (the sum of ‘N in yield,’ ‘Additional harvest N’, ‘Crop total N’, and ‘Crop additional Rubisco N’) for crops with 20% more Rubisco.

The situation in leguminous crops such as soybean which are typically grown without nitrogen fertilization is somewhat different, since here photosynthetically assimilated carbohydrates are used to drive N fixation. From metabolic stoichiometry, it is estimated that the carbon cost of N fixation is 4.13 g of assimilated C per 1 g of fixed N (Holland *et al*., 2023). In common with other legumes, the N content per unit plant mass is considerably higher than in cereals. To maintain this content an estimated 27% of any additional canopy carbon gain would be required. This would lower the potential productivity increase of 8.6% for soybean in the current atmosphere to 6.3%, but would still not require additional N from fertilizer. Each case has assumed that the increase in net crop carbon assimilation translates to an equivalent increase in yield. Many prior studies have shown a degree of sink limitation; specifically an inability of grain crops to utilize additional photosynthate. However, experimental elevation of [CO_2_] around crops, which increases photosynthetic CO_2_ uptake, results in strong yield increases, especially in the most recent cultivar releases of rice and soybean, suggesting breeding has, or could, overcome such limitations (Ainsworth & Long, 2020).

## VIII. Conclusions and future perspectives

Given the strong limitation that Rubisco imposes on maximum photosynthetic rates in both C_3_ and C_4_ crops, it has been and will remain a key target for improvement. As highlighted above, new approaches and tools will accelerate improvement in this key and essential endeavor. However, in terms of technology readiness, both improvement of Rubisco kinetics and introduction of CCMs into crops remain in the discovery phase, Technology Readiness Level (TRL) 1 and 2 as defined (USDA‐NIFA, 2018). Since most improvements here are transgenic, and necessarily so with CCMs, these would require lengthy deregulation under current legislation, suggesting a 20–30 yr time window until these improvements could enter seed systems in volume. Given the major limitation that Rubisco imposes on yield potential, and the large increases in yield per unit land area needed to reach projected future demand, it is critical that these endeavors are accelerated and realized. While a less sophisticated solution, we have shown here that simply increasing Rubisco content can readily be achieved and is able to increase yield, serving as a shorter‐term solution to overcoming the limitations imposed by this central enzyme. It has been reduced to practice in single‐site field trials of both a C_3_ and a C_4_ crop (TRL4), making it ready for wider scale testing (TRL 5) and entry into seed systems on a shorter timescale. Timescales would be further reduced by achieving upregulation through scarless edited mutation of the Rubisco promoter or 5′UTR regions. Finally, we have shown a 20% increase in Rubisco content would not lower crop nitrogen use efficiency and could substantially increase yield per unit land area, making this one potential solution in decreasing pressure to expand the agricultural footprint to meet rise in demand. Further, this approach would be equally applicable to bioenergy and bioproduct feedstocks that can be grown on marginal land unsuitable for food crops, in particular sustainable perennials such as C_4_ Miscanthus and switchgrass or fast growing woody coppice crops such as willows and poplars (Sannigrahi *et al*., 2010; Somerville *et al*., 2010; Baker *et al*., 2022; He *et al*., 2024). Increasing Rubisco provides a means to achieve a moderate increase in photosynthesis and yield in the short term. It is thus not an alternative to the much‐needed larger increases required in the medium and longer term by improving Rubisco kinetics, introducing CCMs and other synthetic opportunities to increase photosynthesis and productivity in crops.

## Competing interests

None declared.

## Disclaimer

The New Phytologist Foundation remains neutral with regard to jurisdictional claims in maps and in any institutional affiliations.

## Supporting information


**Table S1** Calculated cost of synthesizing an additional 20% Rubisco in rice leaves, and expected time to recover this cost via increased photosynthesis.

**Video S1** Animation of the enhancement of leaf net CO_2_ assimilation from additional 20% Rubisco (Δ*A*) in a soybean canopy (*Glycine max* L. *Merr*., LD11‐2170).
Please note: Wiley is not responsible for the content or functionality of any Supporting Information supplied by the authors. Any queries (other than missing material) should be directed to the *New Phytologist* Central Office.
