# Supplementary material for: Increasing Rubisco as a simple means to enhance photosynthesis and productivity now without lowering nitrogen use efficiency
Source: New Phytol. 2024 Dec 17;245(3):951–65. doi: 10.1111/nph.20298 (PMC11711929; doi:10.1111/nph.20298)
Supplement: Supplementary file 1 — Table S1 Calculated cost of synthesizing an additional 20% Rubisco in rice leaves, and expected time to recover this cost via increased photosynthesis. [file NPH-245-951-s002.docx]

New Phytologist Supporting Information

Article title: Increasing Rubisco as a simple means to enhance photosynthesis and productivity now without lowering nitrogen use efficiency

Authors: Coralie E. Salesse-Smith, Yu Wang and Stephen P. Long

Article acceptance date: 5 November 2024

**Supplementary Table 1.** Calculated cost of synthesizing an additional 20% Rubisco in rice leaves, and expected time to recover this cost via increased photosynthesis.

| Parameter | avg | units | Source |
| --- | --- | --- | --- |
| Rubisco as % of leaf mass | 2 | % | (Onoda *et al.*, 2017) |
| Leaf mass per unit land area (rice) | 250 | g m^-2^ | (Cheng *et al.*, 2017) |
| Mass of baseline leaf Rubisco | 5 | g m^-2^ |  |
| Mass of additional 20% leaf Rubisco | 1 | g m^-2^ |  |
| Cost in dry weight lost in synthesizing 1 nmol Rubisco | 1.5 | mg | (Arnold & Nikoloski, 2014) |
| Molecular weight of Rubisco | 550 | kDa | (Falkowski & Raven, 2013) |
| = | 550000 | g mol^-1^ |  |
| = | 0.00055 | g nmol^-1^ |  |
| nmol of an additional 20% leaf Rubisco | 1818.182 | nmol m^-2^ |  |
| Cost in dry weight lost in synthesizing 20% more Rubsico | 2.727273 | g m^-2^ |  |
| net daily assimilation of a rice crop | 30 | g m^-2^ d^-1^ | (Monteith & Moss, 1977) |
| If 20% more Rubisco gives 10% more assimilation the extra assimilation would repay the cost of extra Rubisco in just | 0.909091 | days |  |

**Video S1** **(See other attachment).** Animation of the enhancement of leaf net CO_2_ assimilation from additional 20% Rubisco (Δ*A*) in a soybean canopy (*Glycine max* L. Merr., LD11-2170) throughout the day of 18 August, Champaign, Illinois, United States. The row spacing of the canopy is 76 cm, and plant spacing within the rows 5 cm. Leaf area index is 5.9.

**Supplemental References**

**Arnold A, Nikoloski Z. 2014.** Bottom-up Metabolic Reconstruction of Arabidopsis and Its Application to Determining the Metabolic Costs of Enzyme Production. *Plant Physiology* **165**(3): 1380-1391.

**Cheng T, Song R, Li D, Zhou K, Zheng H, Yao X, Tian Y, Cao W, Zhu Y. 2017.** Spectroscopic Estimation of Biomass in Canopy Components of Paddy Rice Using Dry Matter and Chlorophyll Indices. *Remote Sensing* **9**(4): 319.

**Falkowski PG, Raven JA. 2013.** *Aquatic photosynthesis*: Princeton University Press.

**Monteith JL, Moss CJ. 1977.** Climate and the efficiency of crop production in Britain. *Philosophical Transactions of the Royal Society of London. B, Biological Sciences* **281**(980): 277-294.

**Onoda Y, Wright IJ, Evans JR, Hikosaka K, Kitajima K, Niinemets Ü, Poorter H, Tosens T, Westoby M. 2017.** Physiological and structural tradeoffs underlying the leaf economics spectrum. *New Phytologist* **214**(4): 1447-1463.
