# Supplementary figures and images for: Increasing Rubisco as a simple means to enhance photosynthesis and productivity now without lowering nitrogen use efficiency
Source: New Phytol. 2024 Dec 17;245(3):951–65. doi: 10.1111/nph.20298 (PMC11711929; doi:10.1111/nph.20298)

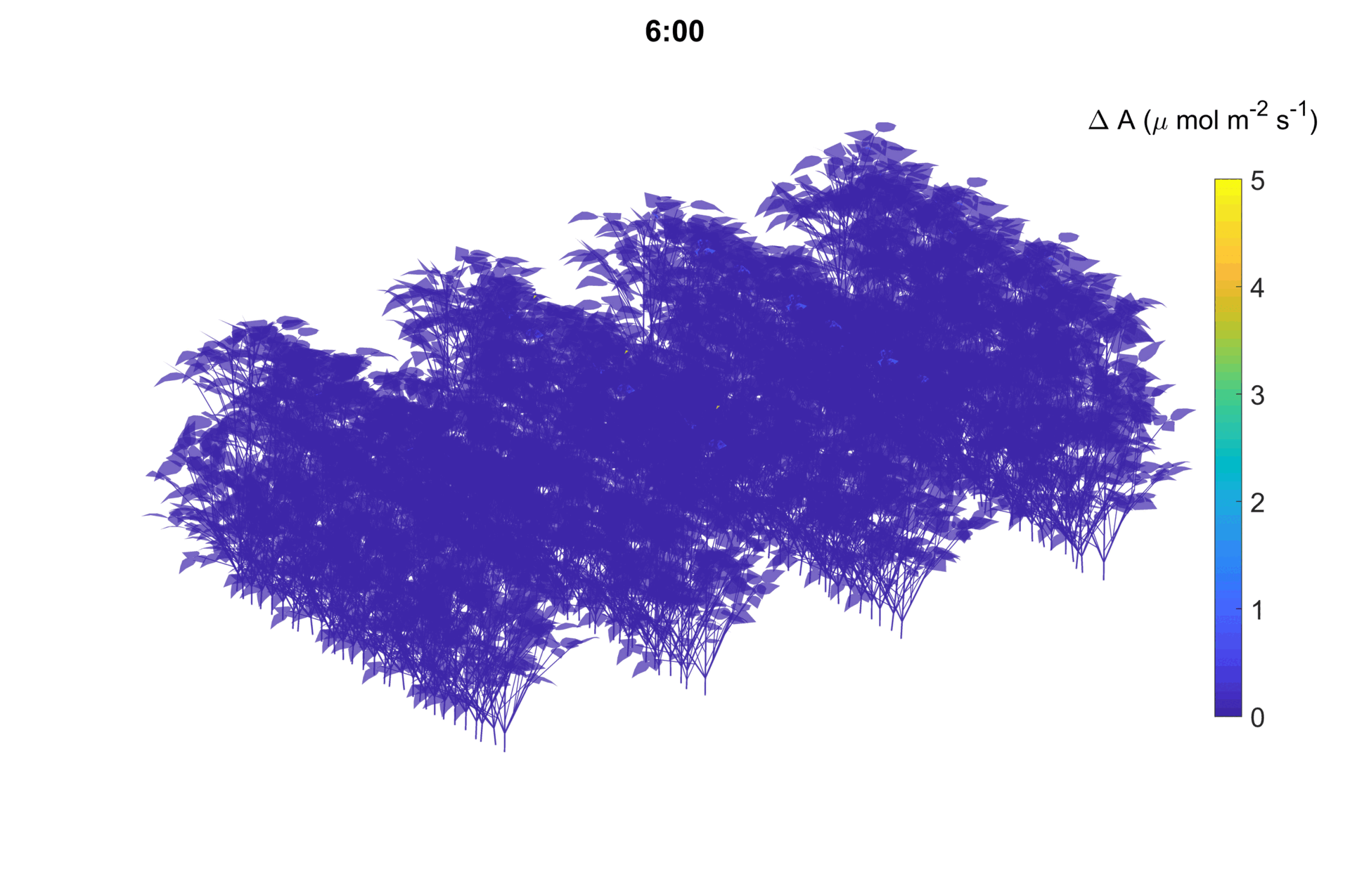

Supplement: Supplementary file 2 — Video S1 Animation of the enhancement of leaf net CO2 assimilation from additional 20% Rubisco (ΔA) in a soybean canopy (Glycine max L. Merr., LD11‐2170). Please note: Wiley is not responsible for the content or functionality of any Supporting Information supplied by the authors. Any queries (other than missing material) should be directed to the New Phytologist Central Office. [file NPH-245-951-s001.gif]
